# Effect of Phenolic Compounds on the Synthesis of Gold Nanoparticles and Its Catalytic Activity in the Reduction of Nitro Compounds

**DOI:** 10.3390/nano8050320

**Published:** 2018-05-10

**Authors:** Elisabete C. B. A. Alegria, Ana P. C. Ribeiro, Marta Mendes, Ana M. Ferraria, Ana M. Botelho do Rego, Armando J. L. Pombeiro

**Affiliations:** 1 Centro de Química Estrutural, Instituto Superior Técnico, Universidade de Lisboa, Av. Rovisco Pais, 1049-001 Lisboa, Portugal; marta.s.mendes@hotmail.com; 2 Chemical Engineering Departament, ISEL-Instituto Superior de Engenharia de Lisboa, Instituto Politécnico de Lisboa, 1959-007 Lisboa, Portugal; 3 CQFM-Centro de Química-Física Molecular and IN-Institute for Nanosciences and Nanotechnologies and IBB-Institute for Bioengineering and Biosciences, Instituto Superior Técnico, Universidade de Lisboa, 1049-001 Lisboa, Portugal; ana.ferraria@tecnico.ulisboa.pt (A.M.F.); amrego@tecnico.ulisboa.pt (A.M.B.d.R.)

**Keywords:** gold nanoparticles, phytochemicals, catalysis, reduction, nitro compounds

## Abstract

Gold nanoparticles (AuNPs) were prepared using an eco-friendly approach in a single step by reduction of HAuCl_4_ with polyphenols from tea extracts, which act as both reducing and capping agents. The obtained AuNPs were characterized by scanning electron microscopy (SEM), transmission electron microscopy (TEM), ultraviolet–visible spectroscopy (UV–vis), and X-ray photoelectron spectroscopy (XPS). They act as highly efficient catalysts in the reduction of various aromatic nitro compounds in aqueous solution. The effects of a variety of factors (e.g., reaction time, type and amount of reducing agent, shape, size, or amount of AuNPs) were studied towards the optimization of the processes. The total polyphenol content (TPC) was determined before and after the catalytic reaction and the results are discussed in terms of the tea extract percentage, the size of the AuNPs, and their catalytic activity. The reusability of the AuNP catalyst in the reduction of 4-nitrophenol was also tested. The reactions follow pseudo first-order kinetics.

## 1. Introduction

Metallic nanoparticles (NPs) are often synthesized using chemical methodologies involving organic and inorganic reducing agents, which can also act as stabilizing agents to avoid the coalescence of the NPs, such as hydrazine, sodium borohydride (NaBH_4_), or *N*,*N*-dimethylformamide [1].

The environmental risks and toxicity associated with these chemicals have led to a growing interest in environmentally friendly and sustainable methods for the synthesis of a variety of metallic NPs with specific sizes and aggregations [2]. Some of these new “green” synthetic processes concern microorganisms, such as bacteria [3,4] or fungi [5], and plant extracts [6,7,8,9,10,11,12].

The use of phytochemicals in the synthesis of NPs, which include leaves, fruits, seeds, and stems, is starting to be developed and presents some advantages over the application of microorganisms in view of the high diversity of plant extracts and the simplicity and low cost of the method [2,6,7,8,9,10].

Gold nanoparticles (AuNPs) are recently gaining a great interest due to their potential application in many fields, such as catalysis, biosensing, photonics, drug-delivery systems, and antimicrobial agents [13,14,15,16,17,18,19]. They also have been used to catalyze electron-transfer and oxidation reactions, specifically glucose oxidation [20], aerobic alcohol oxidation [21], reduction of nitroarenes in aqueous media [22], or CO oxidation and propylene epoxidation [23]. AuNPs produced from vegetable oil in a green reaction medium have also been used in antimicrobial paints [24].

Due to its health benefits and antioxidant properties, the use of black tea [7] for the production of biocompatible AuNPs is being tested in diverse areas such as health and energy. The phytochemicals present in tea show a dual role: as reducing agents to reduce gold and as stabilizers to provide a robust coating on the AuNPs in a one-pot process.

In this work, we used a black tea extract to act as a low-cost reducing and stabilizing agent for AuNPs synthesis, determined the variation of the total polyphenol content (TPC) and the atomic concentration of each element detected by XPS after the AuNPs were produced, and explored the catalytic properties of the AuNPs synthesized via tea extract solutions in simple model reactions; that is, the reduction of various nitro compounds (2- and 4-nitrophenol; 2-, 3-, and 4-nitroaniline, or nitrobenzene) in aqueous solution.

In particular, the reduction of 4-nitrophenol has become the model reaction for the evaluation of the catalytic activity of metallic NPs, namely with Ag [25,26], Au [27,28,29,30,31,32], or Pd [33] metals. The existence of a well-defined absorption band characteristic of this substrate allows to easy screening of the reaction by UV–vis spectroscopy [34,35,36].

## 2. Materials and Methods

### 2.1. Reagents and Instrumentation

All synthetic work was performed in air. The reagents and solvents were obtained from commercial sources and used without further purification or drying. Black tea from Tetley (Yorkshire, UK); 4-nitrophenol (4-NP) (Acros Organics, Morris Plains, NJ, USA); 2-nitrophenol (2-NP) (BDH, St. Louis, MO, USA); nitrobenzene (NB) (Acros); 4-aminophenol (4-AP) (Aldrich, St. Louis, MO, USA); 2-aminophenol (2-AP) (BDH); 2-, 3-, and 4-nitroaniline (2-, 3-, and 4-NA) (Fluka, Bucharest, Romania); 2-, 3-, and 4-phenylenediamine (2-, 3-, and 4-PD) (Fluka, Bucharest, Romania); NaBH_4_ (Acros Organics, Morris Plains, NJ, USA); ascorbic acid (Merck, Darmstadt, Germany); d-glucose (Aldrich, St. Louis, MO, USA); NH_4_Cl (Panreac, Barcelona, Spain); N_2_H_4_ (Aldrich, Missouri, USA); and tetrachloroauric(III) acid (HAuCl_4_∙3H_2_O) (99.99%, Aldrich, St. Louis, MO, USA) were used as received.

The synthesized AuNPs using tea extracts were characterized using UV–vis, XPS, XRD, SEM, EDS, and TEM techniques. UV–visible spectroscopic measurements of the synthesized AuNPs were carried out on a PerkinElmer (Massachusetts, EUA) Lambda 750 UV–visible spectrophotometer. XPS analysis was performed by a XSAM800 spectrometer from KRATOS, Manchester, UK. Details on operation conditions, data acquisition, and treatment were published elsewhere [37], except for the energy reference used to correct the charge shift which was the binding energy of aromatic carbons centered at 284.7 eV. TEM measurements were performed on a transmission electron microscope Hitachi 8100 (Tokyo, Japan) with ThermoNoran light elements EDS detector and digital image acquisition. Morphology and distribution of AuNPs were characterized using a scanning electron microscope (SEM) (JEOL 7001F with Oxford light elements EDS detector and EBSD detector). FTIR (Fourier transform) measurements were carried out on a Bruker Vertex 40 Raman/IR spectrometer (Massachusetts, EUA) in a range from 4000 to 100 cm^−1^.

### 2.2. Preparation of Au Nanoparticles Using Tea Extracts

The preparation of AuNPs using tea extracts followed the method described previously [15]. The tea solutions were prepared by mixing a weighted amount of dried black tea leaves (2 g) with 100 mL of distilled water followed by vigorous stirring for 15 min at room temperature in order to obtain three solutions with different percentages of tea (1, 5, or 10% *w*/*v*). After filtration, 6 mL of each tea solution was transferred to a glass vial and to each of them 0.1 mL of a tetrachloroauric acid (HAuCl_4_∙3H_2_O) solution (0.1 M) was added, with stirring for 20 min at room temperature. The solution changed from pale yellow to red color almost instantaneously, indicating the formation of Au nanoparticles. UV–vis spectroscopy was used to confirm the successful formation of the AuNPs on account of the occurrence of the characteristic surface plasmon resonance (SPR) band of AuNPs (538 nm) in the visible region [22,38,39,40]. X-ray photoelectron spectroscopy analysis confirms the existence of gold and its metallic state in the AuNPs for the 1% tea extract.

### 2.3. Determination of Total Polyphenol Content in Tea by the Folin–Ciocalteu Method

The total polyphenol content (TPC) was determined colorimetrically using the Folin–Ciocalteu phenol reagent and gallic acid as the calibration standard following the reported method [41]. The absorbance of the various reducing media was measured by a UV–vis spectrophotometer at a wavelength of 765 nm, and a calibration curve was constructed with 10 gallic acid standard solutions (12.5–500 mg/L; R^2^ = 0.9996).

### 2.4. Reduction of Nitro Compounds in Aqueous Solution

All catalytic reactions were performed in a standard 3 mL quartz cuvette with a 1 cm path length. Stock aqueous solutions of 4-nitrophenol (0.012 M) and NaBH_4_ (0.1 M) were freshly prepared for each experiment to minimize hydrolysis. Stock aqueous solutions of 2.5 mL of water and 0.5 mL AuNP tea solution (1, 5, or 10%) were prepared. In standard conditions, 0.5 mL of the diluted AuNP solution is placed in the cuvette and 2.5 mL of distilled water is added. To this solution, 100 μL of the nitro compound (2- or 4-nitrophenol; 2-, 3-, or 4-nitroaniline; or nitrobenzene) and finally 50 μL NaBH_4_ solution (0.1 M) was added. The UV–vis absorbance spectra were recorded in the 200–700 nm range at 2 min intervals. The catalytic activity was quantified in terms of conversion of the substrate and turnover frequency (TOF), considering that 2- or 4-aminophenol; 2-, 3-, or 4-phenylenediamine; and aniline are the only reduction products.

## 3. Results and Discussion

### 3.1. Au Nanoparticle Identification

The addition of HAuCl_4_∙3H_2_O to a black tea extract resulted in an almost instantaneous change of color from light yellow to red, which indicates [22,38,39,40] the effectiveness of the Au reduction. As expected, the obtained Au colloid solution exhibits in the UV–vis spectrum the typical [22,38,39,40] absorption band due to the presence of Au nanoparticles (AuNPs), without any significant shift or change in the intensity over time.

**Figure 1:**
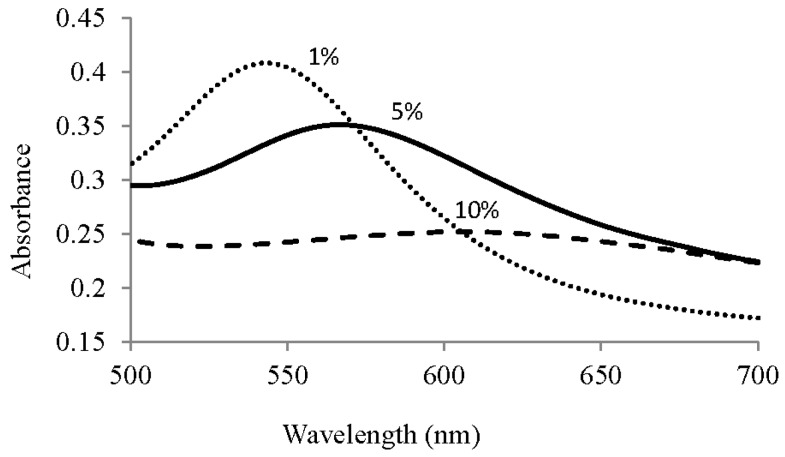
Visible absorbance spectra for AuNPs produced using 1, 5, and 10% tea solutions.

The effect of different concentrations of tea extract (1, 5, and 10% *w*/*v*) solutions on the size and dispersity of the AuNPs was analyzed (onwards, the AuNPs solutions will be named as AuNPs 1% tea, AuNPs 5% tea, and AuNPs 10% tea, respectively). Using different concentrations of tea extract (1, 5, and 10%) enables the variation of the size of the produced nanoparticles and as a result, the colors of their dispersion (Figure 1 and Figure S1).

The surface plasmon resonance (SPR) bandwidth for AuNPs 1, 5, and 10% tea follows the predicted behavior, as it increases with the increasing size of gold nanoparticles [42,43,44]. In fact, the shorter bandwidth for the AuNPs 1% tea solution at 538 nm confirms the smaller size of these NPs (8–24 nm diameter range, see below), relative to the absorption band at 566 nm observed for the AuNPs 5% tea solution or that at ca. 602 nm for the larger-sized AuNPs 10% tea (57–113 nm, see below) (Figure 1).

**Figure 2:**
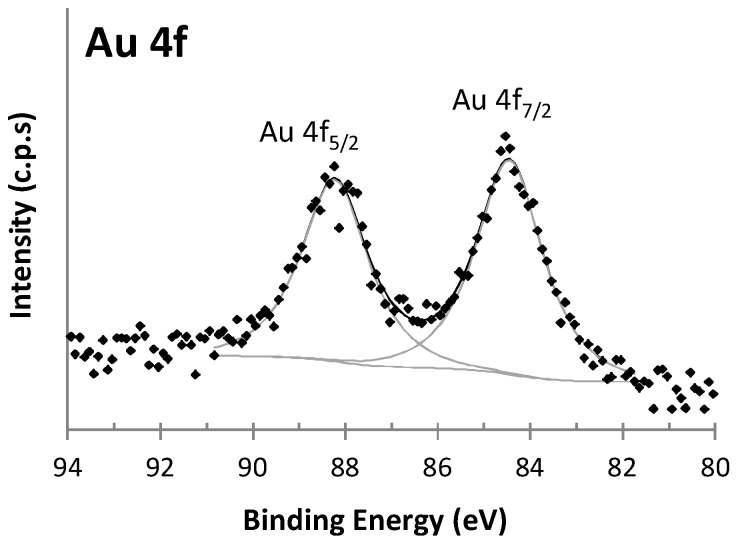
X-ray photoelectron spectroscopy (XPS) Au 4f region for the AuNPs 1% tea.

X-ray photoelectron spectroscopy (XPS) exhibits, in the Au 4f region, a single doublet with the Au 4f_7/2_ component centered at 84.4 ± 0.3 eV (Figure 2). This doublet shows that AuNPs in the 1% tea extract result from the Au^3+^ reduction to Au^0^ and/or Au^+^ and that the precursor no longer exists in the solution [45]. It is worth to notice that although the binding energy for bulky Au^0^ is established to be centered at 83.95 eV [46], drifts to larger or lower binding energies have been reported for very small gold nanoparticles [47]. Besides Au (0.1%), the surface also shows potassium (1.7%), chlorine (2.6%), nitrogen (2.7%), oxygen (29.4%), and carbonaceous species (63.5%), probably due to the tea extract. It is worth to emphasize that in XPS, the atomic percentage depends on the aggregation state. Therefore, for an element in the form of nanoparticles, the atomic percentage is much lower than what it would be if the component atoms were dispersed on the surface. This explains why the gold percentage is the lowest one.

### 3.2. Characterization of AuNPs

**Figure 3:**
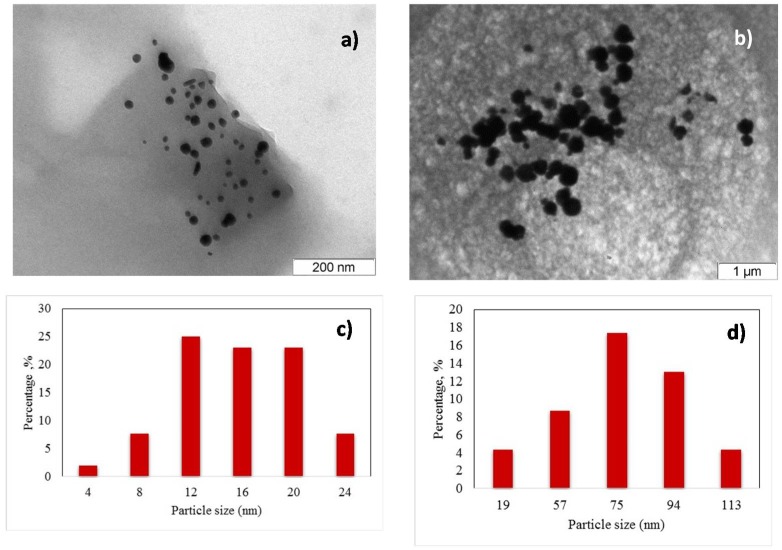
TEM images and size distribution of AuNPs 1% tea (**a**,**c**) and AuNPs 10% tea (**b**,**d**).

Transmission electron microscopy (TEM) was used to determine the details of the microstructure in the samples (Figure 3). The AuNPs produced using a 1% (Figure 3a) or a 10% (Figure 3b) tea extract are spherical and dispersed with nearly no aggregations, which was mainly due to the stabilization effect of the tea extract. The size distribution histograms of the AuNPs (Figure 3c,d) were calculated from the corresponding TEM image in Figure 3a,b by measuring the particles in the image; the average particle sizes being in the 8–24 nm and 57–113 nm ranges, respectively.

**Figure 4:**
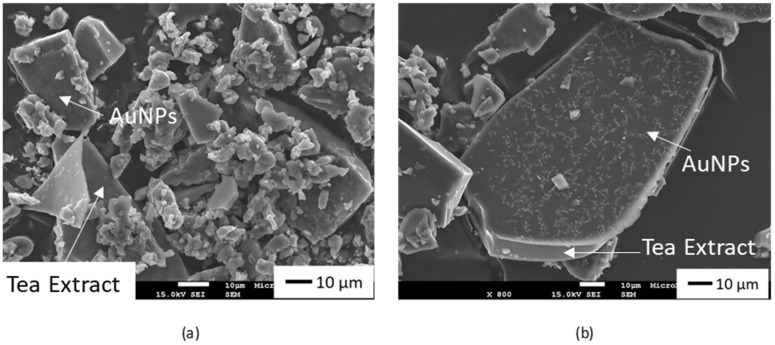
Scanning electron microscopy (SEM) images of (**a**) AuNPs 1% tea and (**b**) AuNPs 10% tea.

The analysis of the AuNPs by energy dispersive X-ray spectroscopy (EDS) confirmed the presence of the signals characteristic of gold, demonstrating the successful immobilization of AuNPs (Electronic Supplementary Information, ESI, Figure S2). Figure 4 shows the scanning electron microscopy (SEM) images of typical samples of AuNPs. Moreover, the combination of SEM and TEM images allows us to conclude that the use of different concentrations of tea extract produced nanoparticles with different sizes. The AuNPs 1% tea are smaller than the AuNPs 10% tea, although both are well dispersed throughout the tea extract. The small white points in Figure 4b are more clear than in Figure 4a due to the diference in the size of AuNPs. For AuNPs 1% tea (Figure 4a), the average size of the AuNPs is smaller than for that of th 10% tea extract. This is in accordance with the size distribution plot produced from the TEM results. The dried tea extract appears as large chunks where the AuNPs are embedded.

FTIR spectra (ESI, Figure S3) were run to identify functional groups on the synthesized AuNPs’ surface. They were recorded for the dried tea extract (before reaction with HAuCl_4_∙3H_2_O) and for the synthetized AuNPs, and showed a great analogy.

Both FTIR spectra displayed a strong and broad signal at 3432 cm^−1^ (hydroxyl group of alcohols or phenols) and a much weaker one at 2926 cm^−1^ (aliphatic CH_2_ asymmetric stretching). The band at 1700–1637 cm^−1^ is due to ν(C=O) of conjugated ketones, aldehydes, quinines, and esters, whereas that at ca. 1406 cm^−1^ is assigned to the deformation of –CH_3_ of alkyl groups from the tea constituents. This could be due to the existence of bioactive molecules in the tea in the absence and in the presence of the AuNPs’ surface.

### 3.3. Determination of Total Polyphenol Content

**Figure 5:**
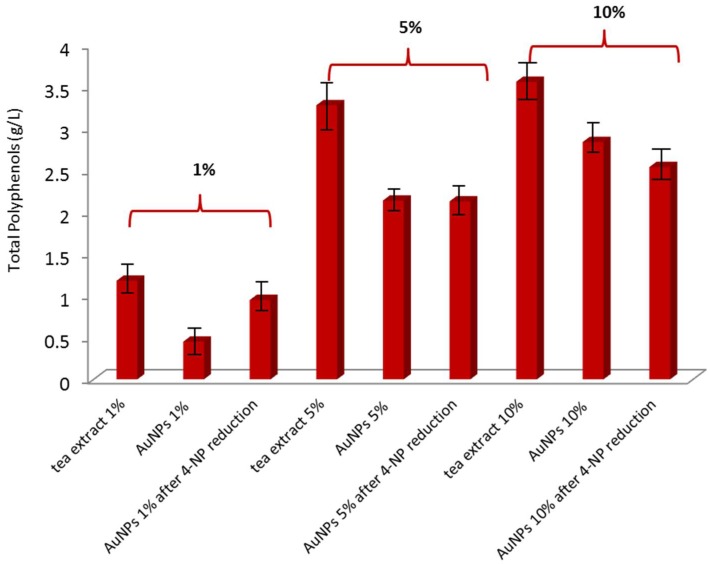
Concentration of total polyphenols in solution (g/L) (4-NP = 4-Nitrophenol).

The different tea extracts (1, 5, and 10%) as well as the produced AuNPs solutions (AuNPs 1% tea, AuNPs 5% tea, and AuNPs 10% tea, respectively), before and after the catalytic reduction of 4-nitrophenol, were analyzed for their total polyphenol content (TPC) (Figure 5). The total polyphenol concentration in the prepared tea stock solutions is 1.18 g/L (for 1% *w*/*v* solution), 3.28 g/L (for 5% *w*/*v* solution), and 3.55 g/L (for 10% *w*/*v* solution), respectively, and as expected, proportional to the amount of tea leaves used to prepare those aqueous solutions (Figure 5, entries 1, 4, and 7).

The polyphenol levels present in the tea extract containing the produced AuNPs (0.46, 2.14, and 2.84 g/L for AuNPs 1% tea, AuNPs 5% tea, and AuNPs 10% tea, respectively) was determined and a reduction of 61, 35, and 20% was observed relative to the initial content (Figure 5, entries 2, 5, and 8). As shown, there is a correlation between the percentage of polyphenols consumed and the size of the produced nanoparticles. As the size of the AuNPs decreased, the amount of polyphenol used for their formation increased (Figure 5). After the catalytic reduction of 4-NP in the presence of AuNPs, the resulting solutions were analyzed and a slight decrease of polyphenol amount was detected for the AuNPs 5% and AuNPs 10% solutions (Figure 5, entries 6 and 9 in comparison with entries 5 and 8, respectively).

Surprisingly, for the AuNPs 1% tea solution, an important increase of polyphenols was detected after the catalytic reduction of 4-NP (Figure 5, entry 3 in comparison with entry 2). This fact can be attributed to (i) conceivable regeneration of polyphenols under catalytic reaction conditions and (ii) possible interference of the small AuNPs with the polyphenol measurements [48].

The surface plasmon resonance wavelength of AuNPs is strongly dependent on the size of individual AuNPs. The enhanced surface plasmon resonance effect of AuNPs is a function of the average size and size distribution. The increasing content of polyphenols after the reaction can be attributed to organic compounds present in the extract and not involved in the characteristic Folin–Ciocalteu reaction, resulting in an overestimation.

### 3.4. Catalytic Studies

The catalytic activity of the prepared AuNPs was investigated for the reduction of various aromatic nitro compounds, namely 2- and 4-nitrophenol (2- and 4-NP); 2-, 3-, and 4-nitroaniline (2-, 3-, and 4-NA); and nitrobenzene (NB) in aqueous medium.

#### 3.4.1. Reduction of 4-Nitrophenol in Aqueous Solution

Catalytic reduction reactions were performed in a standard 3 mL quartz cuvette with a 1 cm path length. The UV–vis absorbance spectra were recorded in the 200–700 nm range at 2 min intervals. A shift of the corresponding absorbance peak at 315 nm to 400 nm occurred immediately after the addition of NaBH_4_. This new peak (at 400 nm) is attributed [27,28,29,30,31,32] to the 4-nitrophenolate ion formed in the presence of NaBH_4_.

**Figure 6:**
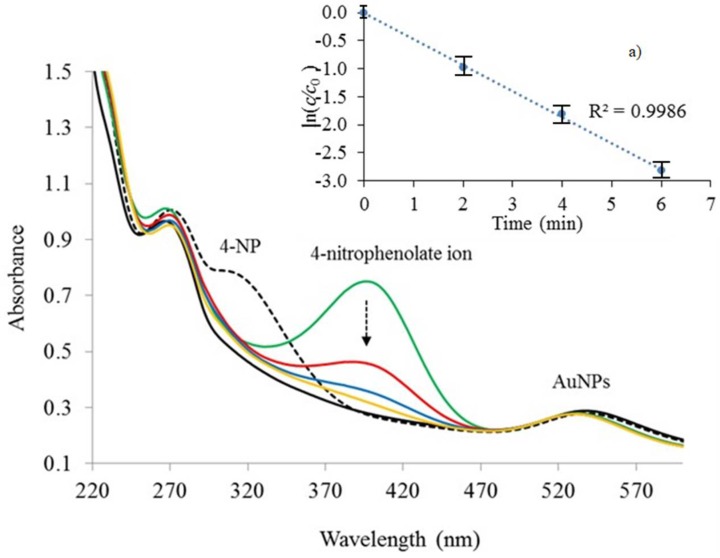
UV–vis spectra over the course of the reduction of 4-nitrophenol (4-NP) by AuNPs 1% tea. Reaction conditions: [AuNPs] = 3.2 × 10^−5^ M; [4-NP] = 3.8 × 10^−5^ M; [NaBH_4_] = 1.6 × 10^−3^ M. Starting AuNPs tea extract aqueous solution (dark solid line); upon addition of 4-NP (dark dashed line); formation of 4-nitrophenolate (green solid line). Reduction reaction after 2 min (red solid line), after 4 min (blue solid line), and after 6 min (yellow solid line). Inset: a) ln(*c*/*c*_0_) versus time (*c* = [4-nitrophenolate]).

The decrease of the intensity of the latter peak readily started in the presence of AuNPs solution and was monitored by UV–vis spectroscopy upon recording the absorbance spectra at 2 min intervals (Figure 6 for 4-NP reduction in the presence of AuNPs 1% tea). The catalytic reduction of 4-nitrophenol (4-NP) to 4-aminophenol (4-AP), widely applied as an industrial chemical and metabolite of common household analgesics such as paracetamol [49], was originally applied by Pal et al. [35] and Esumi et al. [36] to test the catalytic activity of free and immobilized NPs. More recently, this system has been established as a model reaction to evaluate the catalytic performance of different mono- and bimetallic nanoparticles [35,50].

**Scheme 1:**
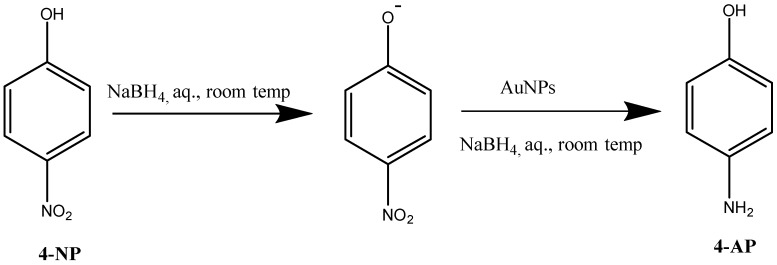
Reduction of 4-nitrophenol (4-NP) to 4-aminophenol (4-AP) by NaBH_4_ catalyzed by AuNPs.

Our study concerned the catalyzed reduction of 4-NP (Scheme 1) in the presence of the synthesized AuNPs, using sodium borohydride (NaBH_4_) as reducing agent. The three types of AuNPs (1, 5, and 10%) were initially tested, and the best results (see below) were obtained for AuNPs 1% tea. Thus, we chose these AuNPs for further studies. 4-Aminophenol (4-AP) is the only reduction product obtained, and the high selectivity was confirmed [27,28,29,30,31,32] by UV–vis spectroscopy.

The effect of different reducing agents other than NaBH_4_ was also studied. Although in the presence of hydrazine, the formation of the intermediate 4-nitrophenolate ion was observed, further reduction to 4-aminophenol was very slight, whereas using other reducing agents such as ascorbic acid, d-glucose, or NH_4_Cl, even the formation of the intermediate ion was not detected.

In this method, the limitation resides in that the concentration of the 4-nitrophenolate ion, and thus the absorbance at 400 nm, is also dependent on other water chemistry parameters, namely the pH of the solution. The pKa of 4-nitrophenol is 7.2 at room temperature, with the 4-nitrophenolate ion as the dominant species at pH > 7.2. The introduction of the reducing agent (sodium borohydride) into the reaction mixture increases the pH and changes the acid–base speciation of 4-nitrophenol (as Scheme 1 and Figure 6 show). To determine if the tea extract has pH-buffering capacity that may gradually decrease the pH of the reaction mixture over time and thus lead to a lowering of the 4-nitrophenolate ion concentration, we monitored the pH of the reaction mixtures over time under the same conditions, and realized that in the case of the reaction in water, the pH increased in the case of using NaBH_4_; however, under the same conditions, but without adding NaBH_4_, the pH of the tea extract solution remains constant (pH ≈ 7). There is a slight increase immediately after the addition of NaBH_4_, but rapidly decreases to the original pH value. From this effect, the decrease in the absorbance at 400 nm is due to the combined effects of the AuNP-catalyzed reduction and changes in pH.

Upon addition of the AuNPs, the electron donor (BH_4_^−^) and electron acceptor (4-nitrophenolate) are both adsorbed on the NP surface and the catalytic reduction of nitrophenolate by BH_4_^−^ starts. In the absence of any catalyst, the formation of the 4-nitrophenolate ion is observed, but no further conversion to 4-aminophenol occurs, and the peak at 400 nm remains unaltered for a long period of time (several days). Thus, AuNPs promote the reduction of 4-nitrophenol by lowering the activation energy of this reaction [51]. In the absence of NaBH_4_, the AuNPs 1% tea catalyst showed no catalytic activity.

The peak due to 4-aminophenol expected at ca. 290 nm (observed for a pure 4-AP solution) could not be clearly observed in our case, since it is masked underneath the absorption band of the tea extract. This absorption band is due to the presence of polyphenols, confirming the strong UV absorbance of the black tea extract.

**Figure 7:**
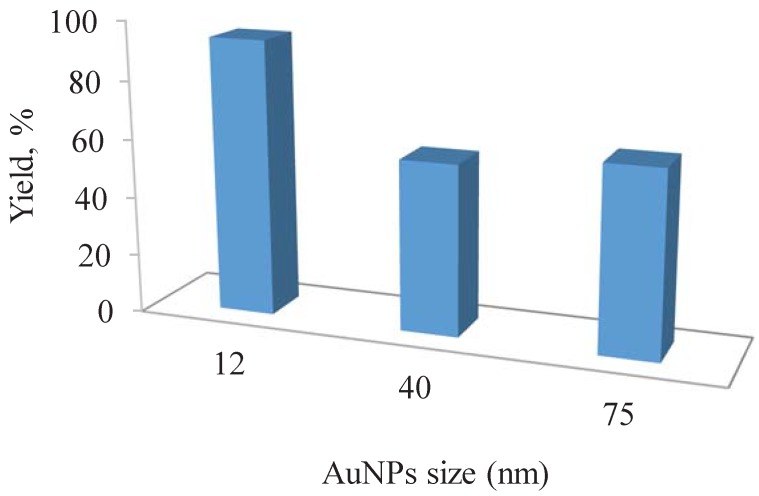
Conversion of 4-nitrophenol (4-NP) to 4-aminophenol (4-AP) as a function of the size of the AuNPs, in the first 6 min of reaction. [AuNPs] = 3.2 × 10^−5^ M; [4-NP] = 3.8 × 10^−5^ M; [NaBH_4_] = 1.6 × 10^−3^ M.

The size of metal NPs plays an important role in catalytic systems [52], and thus the application of different sizes of AuNPs in the reduction of 4-nitrophenol was studied (Figure 7). Although the catalytic activity of the AuNPs usually increases when the size of the particles decreases [52,53,54], an optimal particle size for a particular catalytic system can occur [55,56].

The size of the AuNPs influences the facility with which the substrate (4-NP) and the reducing agent (BH_4_^−^) are adsorbed on the NP surface. Thus, larger nanoparticles show a lower specific surface area and hinder the approach and adsorption of the reactants, lowering their catalytic performance [51]. For instance, for the larger-sized nanoparticles, that is, 75 nm or AuNPs 10% tea, the conversion of 4-NP achieved after the first 6 min of reaction time is ca. 60%, whereas the smaller AuNPs (AuNPs 1% tea), prepared using the less concentrated tea solution, converts 94% of 4-NP (Figure 7).

**Figure 8:**
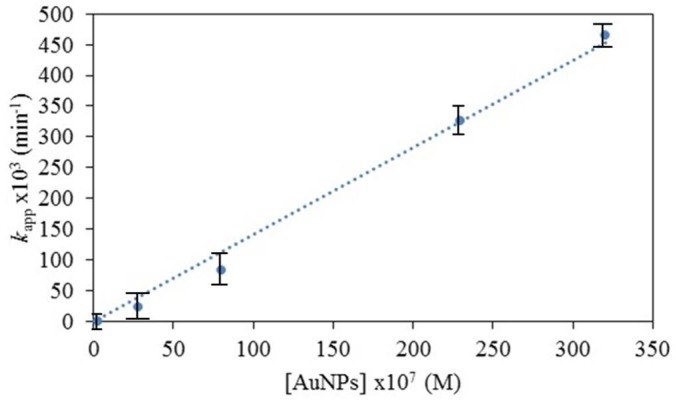
Plot of apparent rate constant *k*_app_ versus concentration of AuNPs (data from Table 1).

The effect of catalyst (AuNPs) concentration on the 4-NP reduction reaction was also studied (Table 1, Figure 8 and Figure S4). The conversion is estimated by the decrease of the band at 400 nm, which is characteristic of the 4-nitrophenolate ion.

For a very low concentration (3 × 10^−7^ M) of AuNPs, almost no conversion of 4-NP to 4-AP was detected after 40 min reaction (entry 1, Table 1). Increasing the catalyst concentration results in a clear rate enhancement. For example, for a 8 × 10^−6^ M catalyst concentration, a conversion of 72% is observed after 18 min (entry 3, Table 1), whereas for 3.2 × 10^−5^ M catalyst, a conversion of 94% is achieved after only 6 min (entry 5, Table 1). Blank tests (in the absence of any NP catalyst) were also performed under common reaction conditions, and no conversion was observed.

Since the reducing agent NaBH_4_ is in a high excess relatively to 4-NP, the reaction is expected to be independent of NaBH_4_ concentration [33,34] and to follow pseudo first-order kinetics (Equations (1) and (2)), where *c* and *c*_0_ are the concentrations of 4-nitrophenolate at time *t* and at *t* = 0, respectively, and ln(*c*/*c*_0_) = ln(*A*/*A*_0_), where *A* and *A*_0_ are the corresponding absorbances of the typical band at 400 nm [33,34,57].

(1) $$-\frac{dc}{dt}=k_{\mathrm{app}}\cdot c$$

(2) ln(cc0)=−kapp⋅t

The value of *k*_app_ is the slope of the plot of ln(*A*/*A*_0_) versus time (see Figure 6a) for the case of AuNPs 1% tea.

A plot of the apparent rate constant versus the concentration of the catalyst is shown in Figure 8, allowing the rate constant (*k*) to be obtained as the slope (1.50 ± 0.05 M^−1^⋅min^−1^).

Furthermore, since the particle size distribution studies indicated that the NPs formed were nearly uniform in size and spherical in shape, we have used the method adopted by Pikeramenou et al. [58] to calculate the number of particles present (ESI, Table S1) in each amount of composite used for catalysis.

This method was applied to calculate the number of surface atoms present in the NPs, which was further used for the calculations of the turnover frequency (TOF) (Equation (3)).

(3) $$TOF=\frac{M}{N_{tS}\cdot t}$$

where *M* is the number of molecules (4-NP) which reacted in the presence of catalyst in time *t* to produce the product (4-AP) and *N_tS_* is the total number of Au surface atoms of the catalyst that are available for the reaction. The details of the method and calculations are included in the Electronic Supplementary Information, ESI.

As expected, the rate of reduction of nitrophenol increases linearly with the catalyst concentration (Figure 8) [59,60,61].

Comparable rates were observed by Panigrahi et al. [39] for the reduction of a series of aromatic nitrocompounds by core-shell nanocomposites (R-Au) bearing, at the surface, well-defined AuNPs of variable sizes (8–55 nm) [39] or by Gangula et al. [57] for the reduction of 4-NP to 4-AP with naturally produced AuNPs (from the stem extract of *Breynia rhamnoides*), although with a larger average size (30 nm), and thus less active, than the AuNPs prepared in our work.

**Figure 9:**
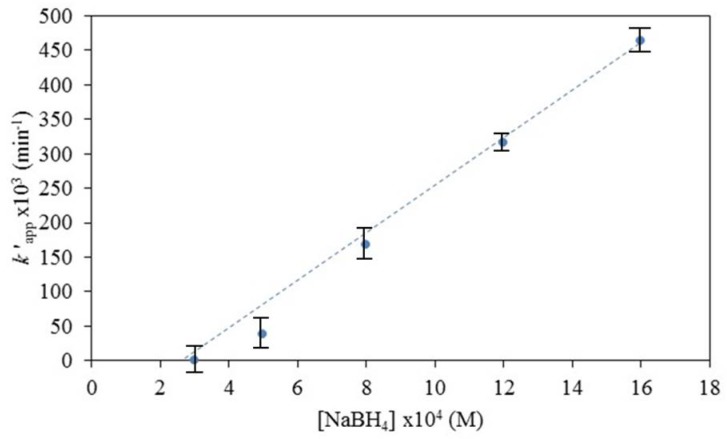
Plot of apparent rate constant *k’*_app_ obtained from Table 2 versus concentration of NaBH_4_ (data from Table 2).

The influence of NaBH_4_ concentration was also investigated for the reduction of 4-NP, for a constant concentration of AuNPs (Table 2, Figure 9 and Figure S5). For the very low NaBH_4_ concentration of 4 × 10^−5^ M, no conversion is observed, even after 40 min of reaction time. Increasing the [NaBH_4_] to 8 × 10^−4^ M allows a 30% conversion to be achieved after the same time (entry 3, Table 2), whereas a conversion of 94% is obtained after only 6 min (entry 5, Table 2) for [NaBH_4_] of 1.6 × 10^−3^ M. No catalyst poisoning seems to occur, since the characteristic strong UV–vis adsorption band of the AuNPs at ca. 538 nm remains with the same intensity. Calculation of the apparent rate constant (*k*’_app_) is performed assuming that the reaction follows a pseudo-first order law (Equations (4) and (5)), where *c* and *c*_0_ are the concentrations of 4-nitrophenolate at time *t* and at *t* = 0, respectively, and ln(*c*/*c*_0_) = ln(*A − A_b_*/*A*_0_ *− A_b_*), where *A*_0_ and *A* are the absorbances at 400 nm at instant *t* = 0 and *t*, respectively, and *A_b_* is the background. All the values presented show the correction due to the background absorbance at 400 nm.

(4) $$-\frac{dc}{dt}={k^{\prime }}_{\mathrm{app}}\cdot c$$

(5) ln(cc0)=−k′app⋅t

Higher concentrations of NaBH_4_ for the 4-nitrophenol-catalyzed reduction induce faster kinetics from zero- to first-order kinetics (ESI, Figures S5 and S6).

The rate of reaction also increases with the concentration of sodium borohydride, as observed by other authors [34,39,59].

#### 3.4.2. Reduction of Other Nitro Compounds

The catalytic activity of the AuNPs 1% was also evaluated for the reduction of 2-nitrophenol (2-NP), nitroanilines (NA), and nitrobenzene (NB), with NaBH_4_ as the reducing agent (Table 3, Figures S7–S9).

In all cases, the conversion is more efficient for the compounds with the nitro group in the 4-position in comparison with those in the 2- and 3-positions (Table 3). For instance, for the reduction of 4-NA to 4-phenylenediamine (4-PD), 77% conversion is achieved shortly after 8 min (entry 4, Table 3); while for 2- and 3-NA (entries 2 and 3, Table 3), the conversion is 40% and 15%, respectively.

The product of 4-NA reduction, 4-phenylenediamine (4-PD), is an important member of aminobenzenes, with applications in dyes [62] and rubber antioxidants [63], and can also be used as a precursor to aramid textile fibers, thermoplastics, and cast elastomers [64]. In industry, the preparation of 4-PD involves the catalytic hydrogenation of 4-NA [64]. Thus, developing an economic and simple route to its preparation is of great interest.

The intensity decrease of the absorbance peak at 380 nm, due to 4-NA, during the reduction (Figure S8) is accompanied by the appearance of a new one at 308 nm, corresponding to the formation of 4-PD. Another characteristic peak of 4-PD at 238 nm is not visible due to the overlap with a tea extract absorption band. Initially, no other products were detected, but after a longer period of reaction, reoxidation of the amino group of 4-PD to give benzoquinone and/or azobenzene may occur (with appearance of a new absorption band at ca. 486 nm) [65].

The reduction of nitrobenzene (NB), since the classical work by Haber [66], is a well-studied reaction. In the presence of AuNPs, the reduction proceeds only along the so-called direct route, via nitrosobenzene and phenylhydroxylamine [67,68,69].

In our catalytic system, nitrobenzene is less reactive than 4-NP against NaBH_4_ in the presence of the same amount of AuNPs (3.2 × 10^−5^ M), and provides, after 8 and 14 min (ESI, Figure S9), ca. 61% (entry 5, Table 3) and 70% conversion, respectively. After 40 min, the conversion is not yet complete.

#### 3.4.3. Recycling Studies for AuNPs in 4-NP Reduction

**Figure 10:**
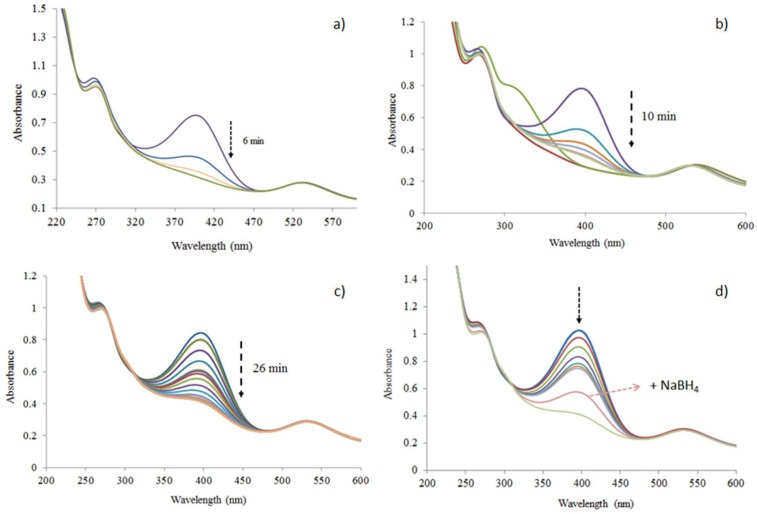
Reutilization of AuNPs 1% tea as the catalyst in the reduction of 4-NP. (**a**) First addition of 4-NP; (**b**) second addition of 4-NP; (**c**) third addition of 4-NP; (**d**) fourth addition of 4-NP and subsequent addition of NaBH_4_. All additions were in identical conditions to those of entry 5, Table 1.

To check the possibility of reutilization and recycling of our AuNPs as the catalyst in the reduction of nitro compounds, we repeated the reduction experiments of 4-NP via two different procedures (I and II). In the first one (I), 4-NP reduction was performed through sequential addition of new portions of substrate (after the previous one had been consumed) to an aqueous solution containing NaBH_4_ in excess and AuNPs 1% tea (Figure 10). The AuNPs become less efficient upon each new addition of 4-NP. However, even after the third addition of substrate (Figure 10c) and in the presence of the initial AuNPs catalyst, conversion of 4-NP to 4-AP is still observed, although taking longer to almost complete the transformation (26 min in comparison with the 10 min of the second addition (Figure 10b) or the 2 min for the first one (Figure 10a)).

After the fourth addition of substrate (Figure 10d), it was found that the conversion stagnated after 12 min. However, upon addition of a new amount of the reducing agent NaBH_4_, a full descent of the characteristic band of the 4-nitrophenolate was detected, demonstrating the complete conversion of the substrate and that the AuNPs 1% are still active and therefore available for recycling.

The recycling of the AuNPs 1% catalyst was also studied (procedure II), by separating the AuNPs from the reaction mixture (by centrifugation) after a first reduction reaction (Figure 10a).

**Figure 11:**
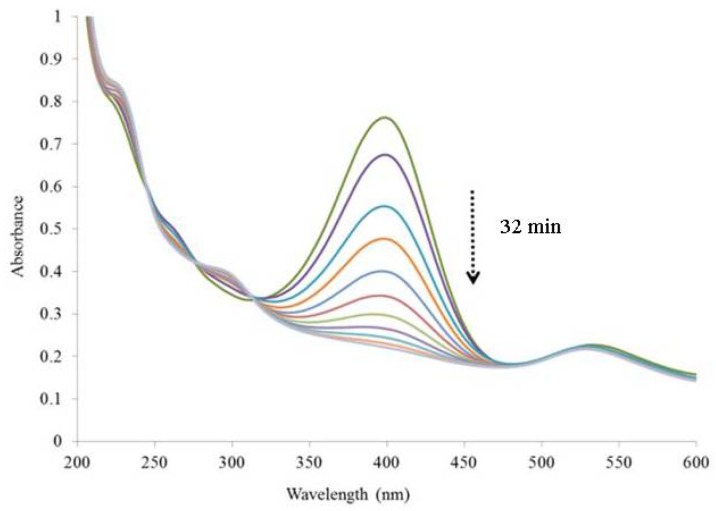
Recycling of AuNPs 1% tea (separated by centrifugation) as the catalyst in the reduction of 4-NP.

The deposited AuNPs were washed with distilled water, and new portions of 4-NP and reducing agent were added (Figure 11). The volume was completed with distilled water.

The reduction of 4-NP takes 32 min to be completed, whereas in the first method, it happens after 10 min (Figure 10b). It was also found that since the tea solution was removed after centrifugation, the characteristic band of 4-AP at 290 nm is now visible and not masked with tea extract bands. Moreover, the tendency to form agglomerates using the last method could lead to the reduced reactivity and stability of these nanoparticles [70]. In fact, in the first cycles, a shift from 538 nm to 630 nm in the characteristic band of AuNPs was observed, indicating that the size of the nanoparticles increased due to aggregation. With the progression of the reaction, a new shift in the band, now to lower wavelengths, is observed. The change could be due to the reaction of the reducing agent with AuNPs, promoting their decrease in size.

The heterogeneous nature of the catalytic process was also proven as follows. After the first cycle, the AuNPs were separated from the final reaction mixture by centrifugation (Figure 11). The resulting solution was also tested for the reduction of 4-NP, and no activity was observed. Moreover, the AuNP characteristic absorption band was not detected.

The use of a heterogeneous nanocatalyst produced by an eco-friendly procedure is still an underdeveloped field, and a comparison with other related cases shows that our catalyst is more active than a similar one supported in multiwalled carbon nanotubes [71], where the reduction of 4-NP was not complete even after 2 h. In the case of the AuNPs synthesized from the stem extract of *Breynia rhamnoides* [57], 90% conversion was observed after 7 min, whereas over 94% conversion was observed in 6 min with the AuNPs 1% tea produced in this work.

## 4. Conclusions

The results show that AuNPs were successfully synthesized in a single step process upon reduction of HAuCl_4_∙3H_2_O by polyphenols and flavonoids present in black tea, without using a surfactant. These gold nanoparticles were utilized as efficient heterogeneous catalysts for the reduction of nitro compounds. High conversions into the corresponding amino-products were obtained, no particle aggregation occurred during the catalytic process, and the AuNP catalyst can be easily reused and recycled. The black tea method used for the synthesis of nanoparticles is quite simple and environmentally benign, and the work shows that the AuNPs obtained thusly act as good catalysts for nitro reductions, thus promoting greener and safer methodologies for such reactions.

## Figures and Tables

**Table 1:** Apparent rate constant, conversion, and TOF for the reduction of 4-NP to 4-AP with variable concentrations of AuNPs *^a^*.

Entry	[AuNPs] (M)	*k*_app_ × 10^3^ (min^−1^)	Conversion *^b^* (%)	TOF *^b,c^* (h^−1^)	Time *^d^* (min)	Conversion (%)	TOF *^c^* (h^−1^)
1	3.0 × 10^−7^	0.1	0.0	0.0	40	0.4	5.1
2	2.8 × 10^−6^	22.2	0.0	0.0	20	9.3	25.8
3	8.0 × 10^−6^	82.8	22.7	72.8	18	72.1	77.1
4	2.3 × 10^−5^	325	64.3	71.8	14	93.1	45.2
5	3.2 × 10^−5^	464	94.0	75.4	6	94.0	75.4

*^a^* Reaction conditions: [4-NP] = 3.8 × 10^−5^ M, [NaBH_4_] = 1.6 × 10^−3^ M, catalyst = AuNPs 1% tea. *^b^* 6 min reaction time. *^c^* TOF = TON per hour (estimated from Equation (3), see ESI, Table S1); TON = number of moles of product per mol of metal catalyst. *^d^* Time beyond which no further conversion was significant. *k_app_* = apparent rate constant.

**Table 2:** Reduction of 4-NP to 4-AP with variable concentrations of NaBH_4_ *^a^*.

Entry	[NaBH_4_] (M)	*k’*_app_ × 10^3^ (min^−1^)	Conversion *^b^* (6 min) (%)	Time *^d^* (min)	Conversion (%)	TOF *^c^* (h^−1^)
1	3.0 × 10^−4^	1.0	0.0	40	2.4	8.5
2	5.0 × 10^−4^	38.1	2.4	40	18.5	43.9
3	8.0 × 10^−4^	168	22.8	40	30.2	130
4	1.2 × 10^−3^	315	55.9	14	88.2	76.3
5	1.6 × 10^−3^	464	94.0	6	94.0	127

*^a^* Reaction conditions: [4-NP] = 3.8 × 10^−5^ M and [AuNPs 1%] = 3.2 × 10^−5^ M. *^b^* 6 min reaction time. *^c^* TOF = TON per hour (estimated from Equation (3)); TON = number of moles of product per mol of metal catalyst. *^d^* Time beyond which no further conversion was significant.

**Table 3:** Reduction of various substrates using AuNPs 1% tea as catalyst *^a^*.

Entry	Substrate	Product	*k*_app_ × 10^2^ (min^−1^)	Time (min)	Conversion (%)	TOF *^b^* (h^−1^)
1	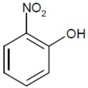	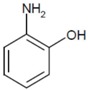	23.9	8	80	61.1
	(2-NP)	(2-AP)				
2	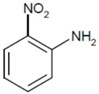	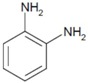	17.3	8	40	30.5
	(2-NA)	(2-PD)				
3	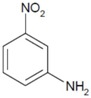	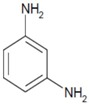	2.5	8	15	11.4
	(3-NA)	(3-PD)				
4	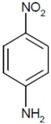	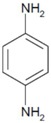	37.8	8	77	58.7
	(4-NA)	(4-PD)				
5	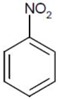	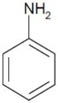	4.6	8	61	46.5
	(NB)	(NA)				

*^a^* Reaction conditions: [AuNPs 1%] = 3.2 × 10^−5^ M; [substrate] = 3.8 × 10^−5^ M; [NaBH_4_] = 1.6 × 10^−3^ M at 25 °C. *^b^* TOF = TON per hour; TON = number of moles of product per mol of metal catalyst.

## Supplementary Materials

The following are available online at http://www.mdpi.com/2079-4991/8/5/320/s1, Figure S1: Gold nanoparticles (AuNPs 1% tea, AuNPs 5% tea and AuNPs 10% tea) prepared by addition of [HAuCl_4_.3H_2_O] = 1.0 × 10^−1^ M to black tea extracts with different concentrations (1, 5 or 10%), Figure S2: EDS image and spectrum corresponding to a selected area for (a) AuNPs 1% tea and (b) AuNPs 10% tea; (c) EDS image and spectrum corresponding to a selected area for tea extract, Figure S3: FTIRS of (a) 1% tea stock solution; (b) AuNPs 1% tea, Figure S4: UV-Vis spectra for the reduction of 4-NP to 4-AP with variable concentrations of AuNPs. Reaction conditions: [4-NP] = 3.8 × 10^−5^ M and [NaBH_4_] = 1.6 × 10^−3^ M, Figure S5: Sucessive UV-Vis spectra for the reduction of 4-nitrophenol (4-NP) by AuNPs 1% tea with different concentrations of reducing agent [NaBH_4_], Figure S6: Reduction of 4-NP to 4-AP with variable concentrations of NaBH_4_, Figure S7: UV-Vis spectra run along the reduction of 2-nitrophenol (2-NP) by AuNPs 1% tea, Figure S8: Sucessive UV-Vis spectra for the reduction of 4-nitroaniline (4-NA) by AuNPs 1% tea, Figure S9: Sucessive UV-Vis spectra for the reduction of nitrobenzene by AuNPs1% tea, Table S1: Calculation of the number of AuNPs, of the number of Au surface atoms and of turnover frequency.
