# Supplementary material for: Effect of Phenolic Compounds on the Synthesis of Gold Nanoparticles and Its Catalytic Activity in the Reduction of Nitro Compounds
Source: Nanomaterials (Basel). 2018 May 10;8(5):320. doi: 10.3390/nano8050320 (PMC5977334; doi:10.3390/nano8050320)
Supplement: Supplementary file 1 [file nanomaterials-08-00320-s001.pdf]

Electronic Supplementary Information

Effect of phenolic compounds on the synthesis of gold nanoparticles and its catalytic activity

Elisabete C.B.A. Alegria,[a,b,*] Ana P.C. Ribeiro,[a*] Marta Mendes,[a,b] Ana M. Ferraria,[c] Ana M. Botelho do Rego,[c] Armando J.L. Pombeiro[a,*]

[a]Centro de Química Estrutural, Instituto Superior Técnico, Universidade de Lisboa, Av. Rovisco Pais, 1049-001 Lisboa, Portugal.
[b]Chemical Engineering Departament, ISEL-Instituto Superior de Engenharia de Lisboa, Instituto Politécnico de Lisboa, 1959-007 Lisboa, Portugal.
[c] CQFM-Centro de Química-Física Molecular and IN-Institute for Nanosciences and Nanotechnologies and IBB-Institute for Bioengineering and Biosciences, Instituto Superior Técnico, Universidade de Lisboa, 1049-001 Lisboa, Portugal.

*Corresponding authors

1. Synthesis of AuNPs

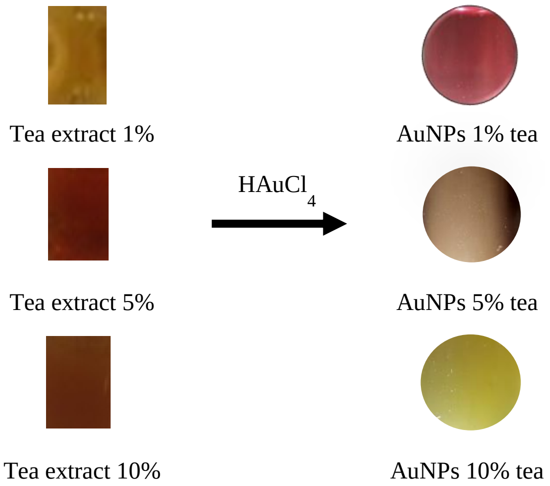

Figure S1 - Gold nanoparticles (AuNPs 1% tea, AuNPs 5% tea and AuNPs 10% tea) prepared by addition of [$HAuCl_4.3H_2O$] = $1.0x10^{-1}$ M to black tea extracts with different concentrations (1, 5 or 10 %).

2 . Characterization of AuNPS

Peaks for Cu and C are from the grid used, and the peaks for K, Mg and O correspond to the capping over the AuNPs (Figures S2a and S2b) for all the tested tea extracts. A sample of the tea stock solution was also studied and it was confirmed that the observed extra peaks are derived from the dried tea (Figure S2c).

AuNPs1% tea

a)

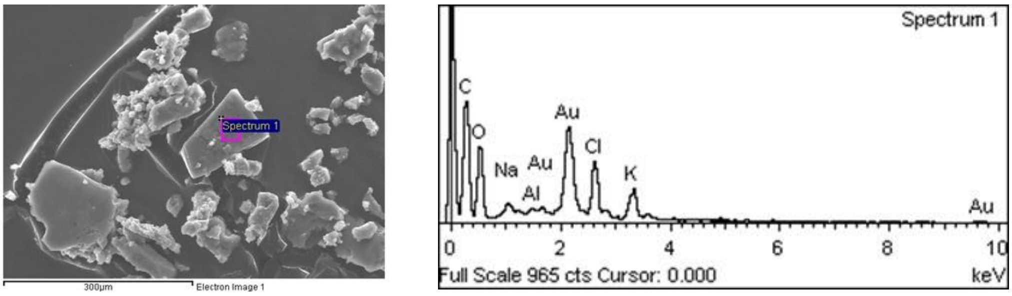

AuNPs10% tea

b)

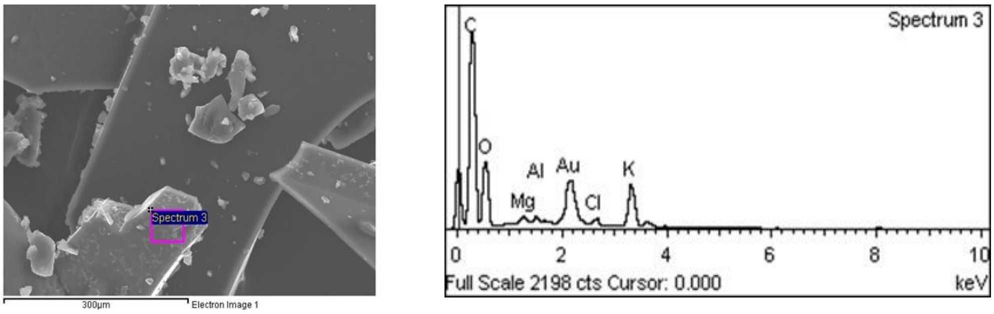

Tea solution

c)

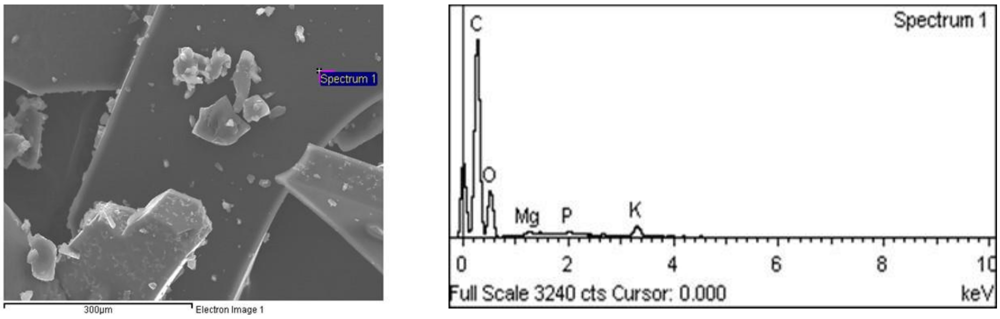

Figure S2 – EDS image and spectrum corresponding to a selected area for (a) AuNPs1% tea and (b) AuNPs 10% tea; (c) EDS image and spectrum corresponding to a selected area for tea extract. The white square is to show the target area for each analysis.

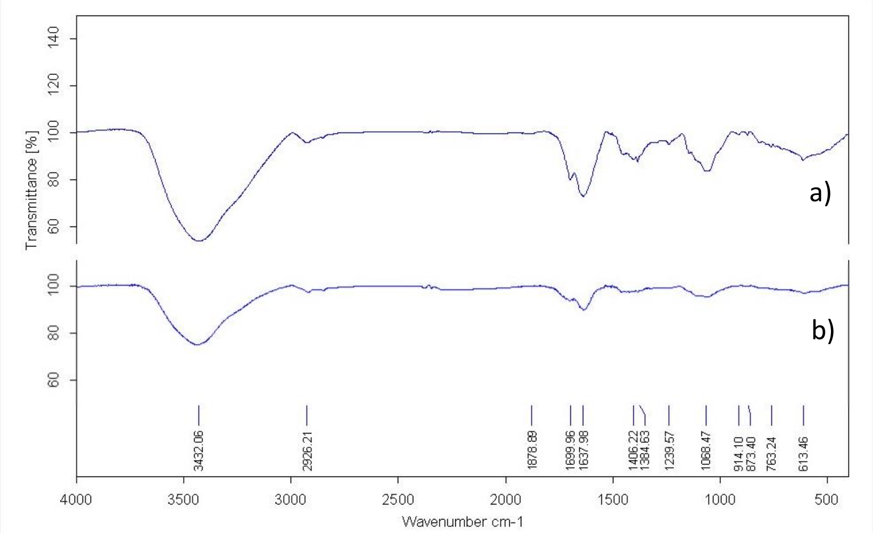

Figure S3 - FTIRS of (a) 1% tea stock solution; (b) AuNPs1% tea.

3 . UV studies for AuNPS

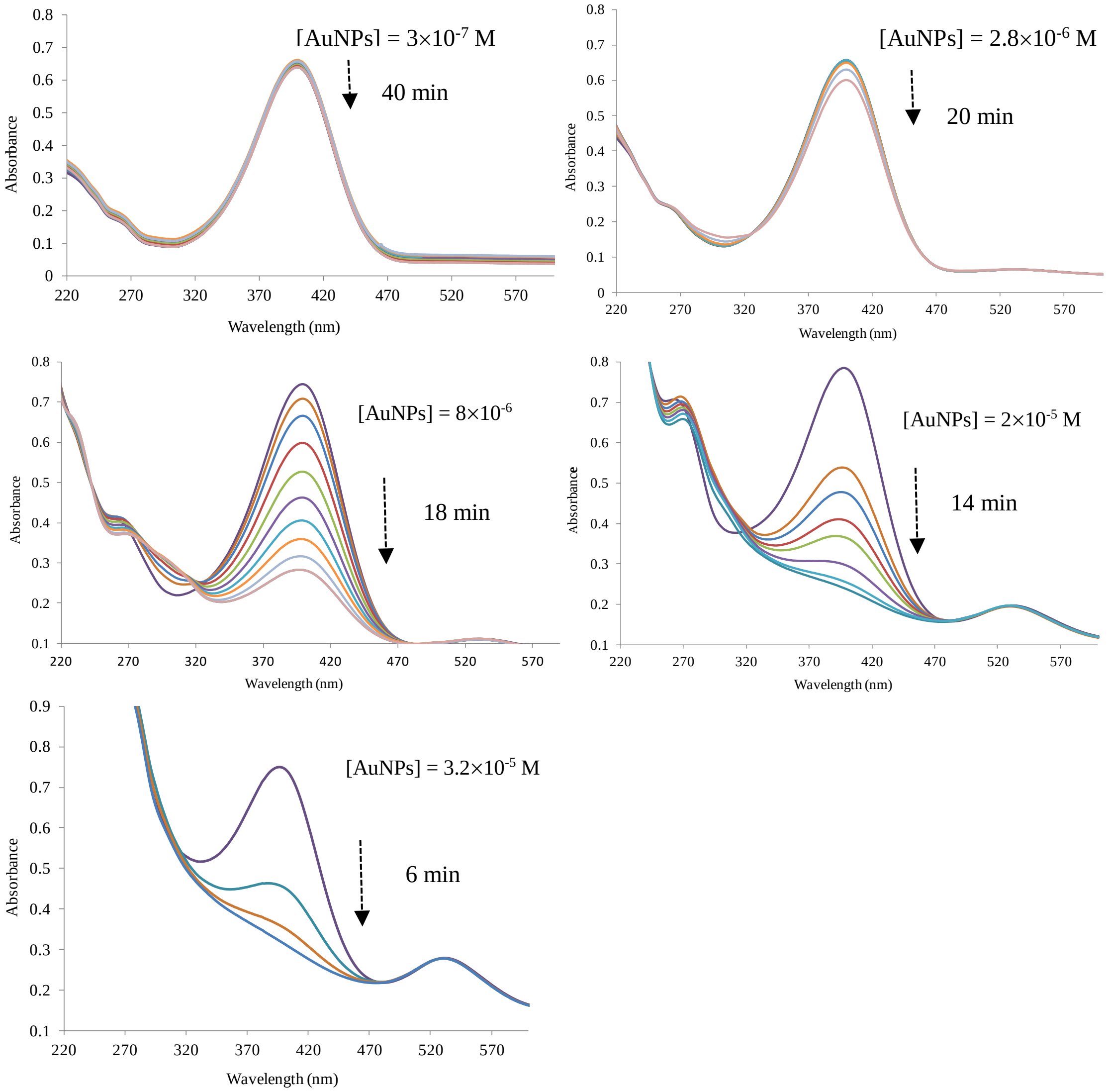

Figure S4 – UV-Vis spectra for the reduction of 4-NP to 4-AP with variable concentrations of AuNPs. Reaction conditions: [4-NP] = 3.8×10^{-5} M and [$NaBH_4$] = 1.6×10^{-3} M.

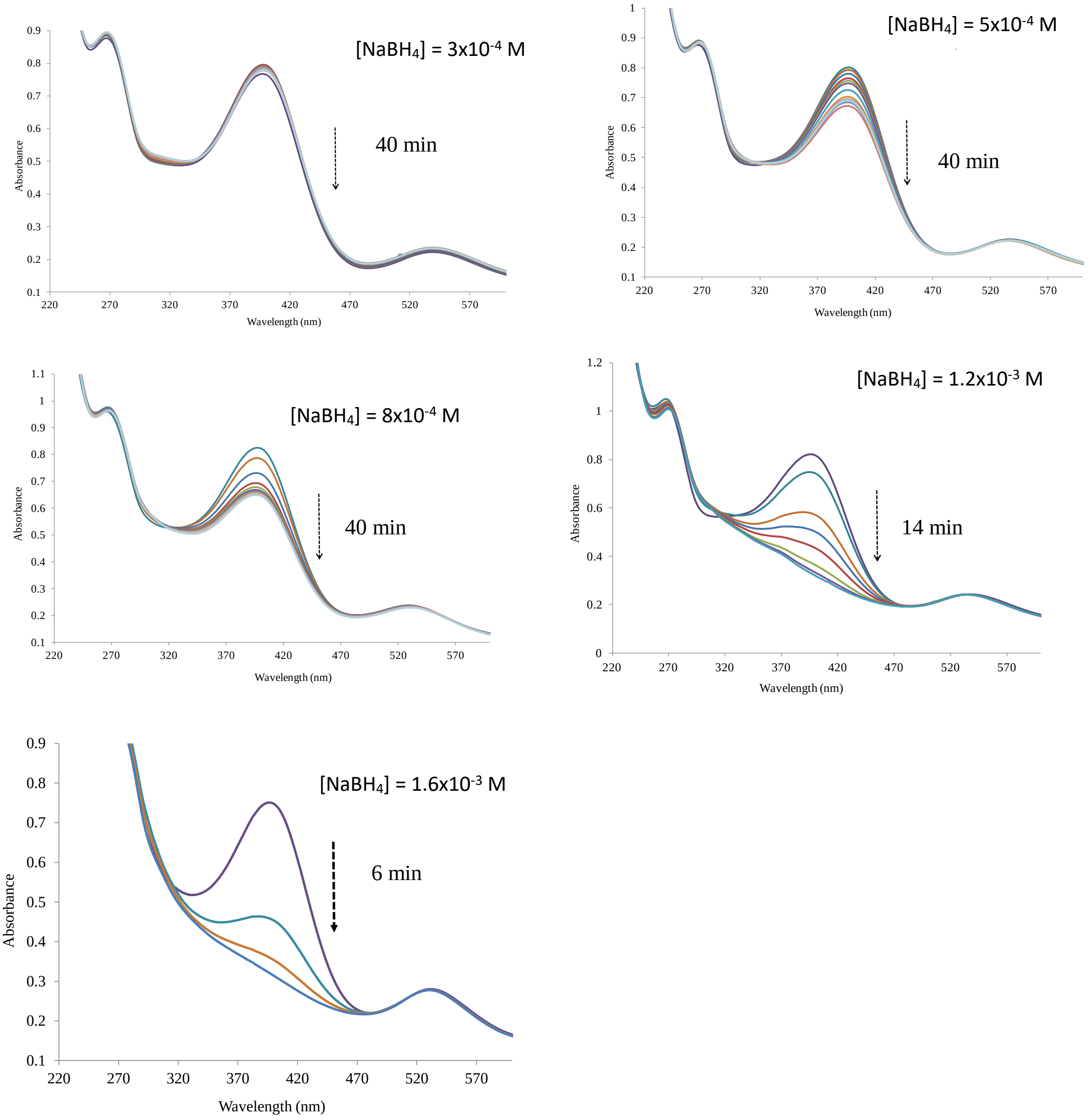

Figure S5 - Sucessive UV-Vis spectra for the reduction of 4-nitrophenol (4-NP) by AuNPs1% tea-with different concentrations of reducing agent [$NaBH_4$].

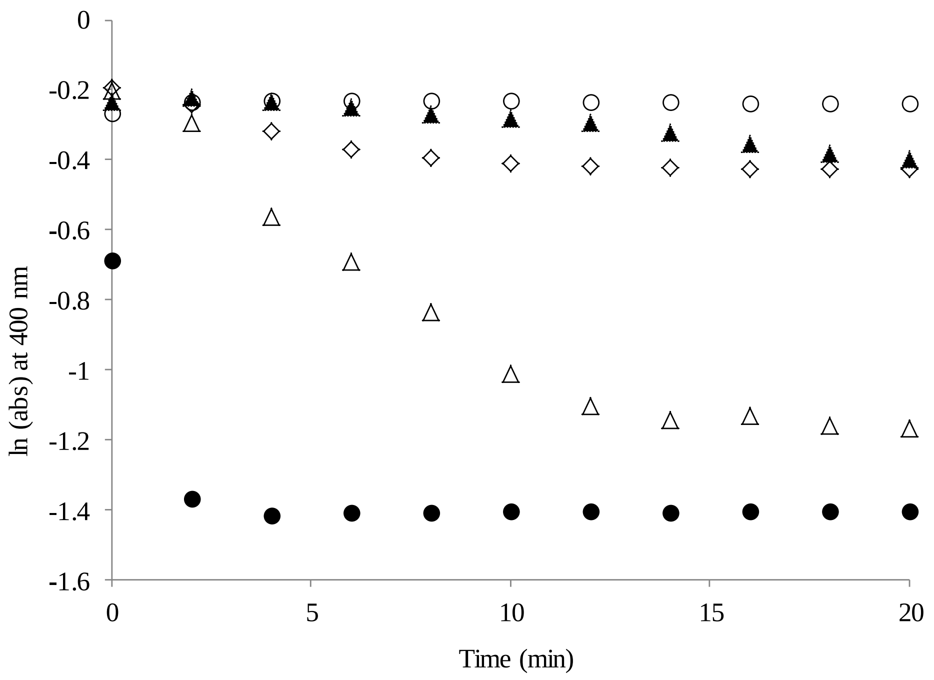

Figure S6 - Reduction of 4-NP to 4-AP with variable concentrations of $NaBH_4$. ○ - $[NaBH_4] = 3\times10^{-4}$ M; ▲ - $[NaBH_4] = 5\times10^{-4}$ M; ◊ - $[NaBH_4] = 8\times10^{-4}$ M; △ - $[NaBH_4] = 1.2\times10^{-3}$ M; ● - $[NaBH_4] = 1.6\times10^{-3}$ M.

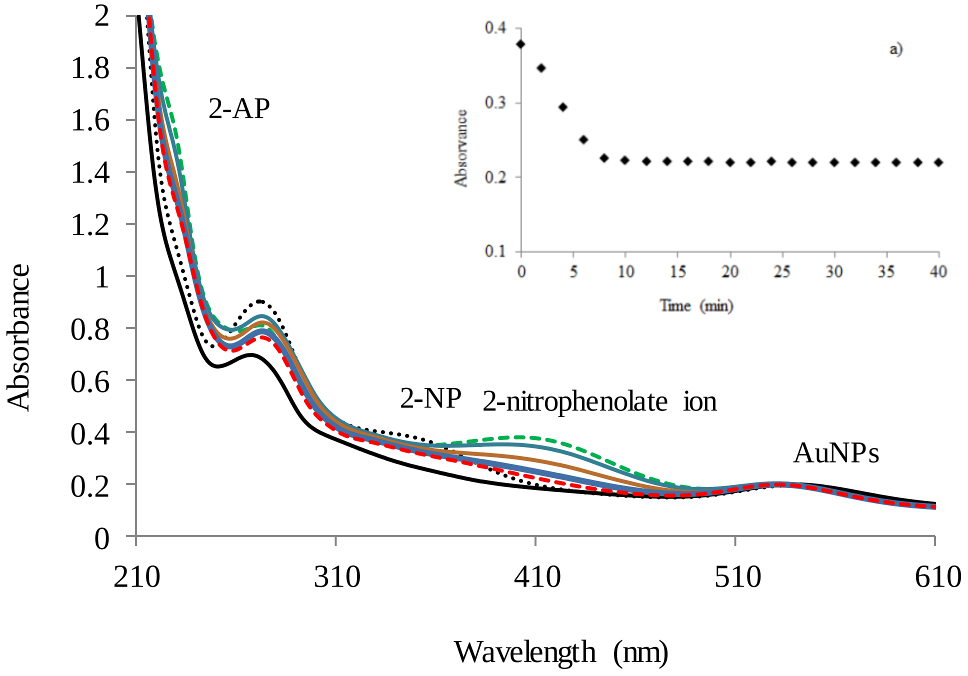

Figure S7 - UV-Vis spectra run along the reduction of 2-nitrophenol (2-NP) by AuNPs 1% tea. Reaction conditions: [AuNPs 1% tea] = 3.2×10^{-5} M; [2-NP] = 3.8×10^{-5} M; [$NaBH_4$] = 1.6×10^{-3} M. Starting AuNPs tea extract aqueous solution (dark solid line); upon addition of 2-NP (dark dashed line); formation of 2-nitrophenolate (red dash line); after 8 min. reduction (conversion of 2-NA in 2-PD) (red dashed line). a) Absorbance at 415 nm *vs*. time.

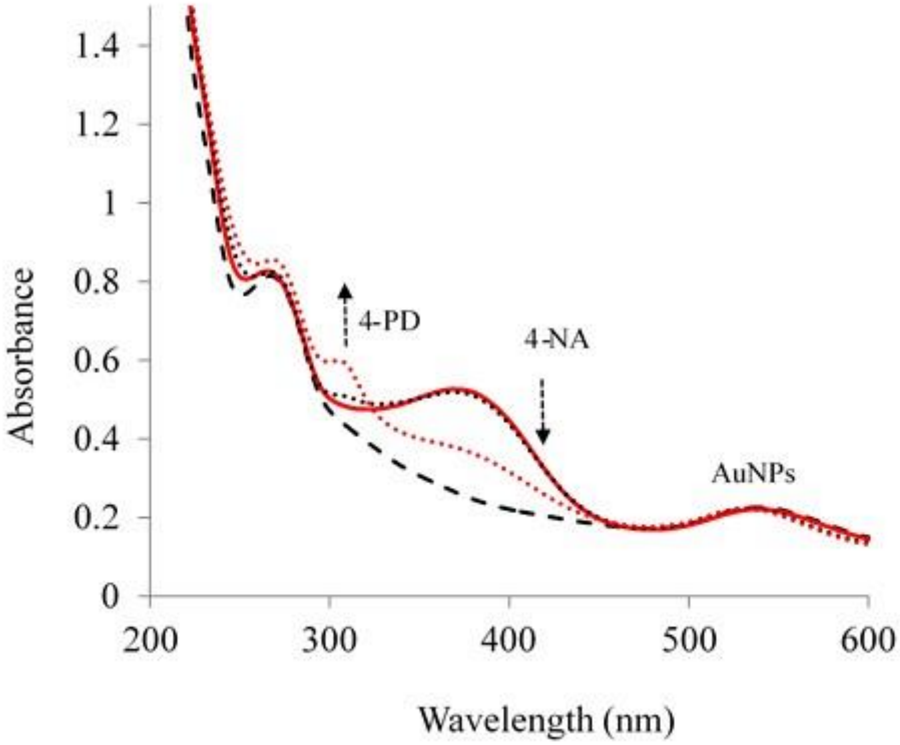

Figure S8 - Sucessive UV-Vis spectra for the reduction of 4-nitroaniline (4-NA) by AuNPs 1% tea. Reaction conditions: [AuNPs1% tea] = 3.2×10^{-5} M; [4-NA] = 3.8×10^{-5} M; [$NaBH_4$] = 1.6×10^{-3} M. Starting AuNPs tea extract aqueous solution (dark dashed line); upon addition of 4-NA (red solid line); upon addition of $NaBH_4$ redutor (dark solid line) and after 2 min. reduction (conversion of 4-NA in 4-PD) (red dashed line).

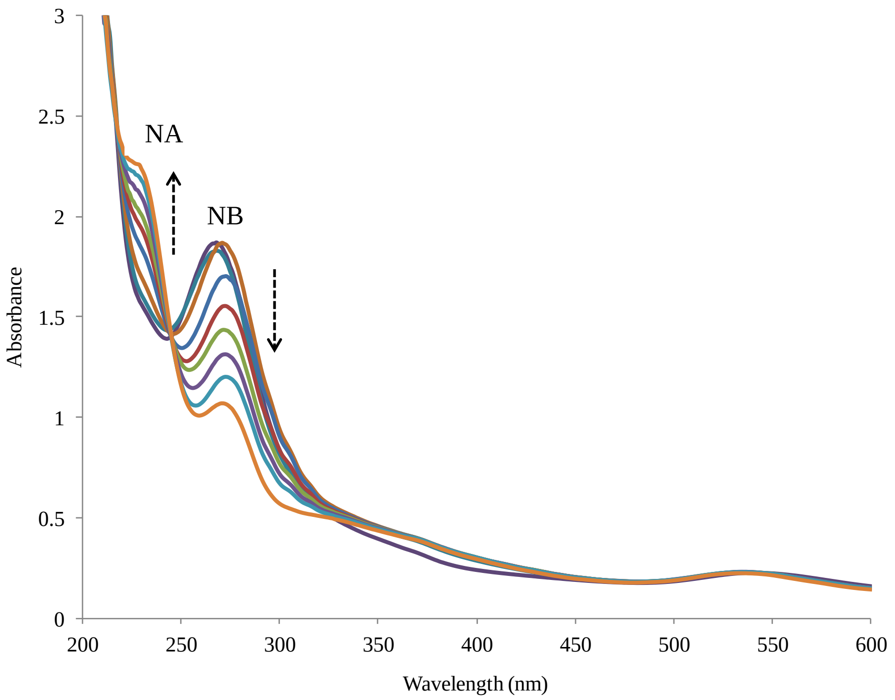

Figure S9 - Sucessive UV-Vis spectra for the reduction of nitrobenzene by AuNPs1% tea. Reaction conditions: [AuNPs1% tea] = 3.2×10^{-5} M; [NB] = 3.8×10^{-5} M; [$NaBH_4$] = 1.6×10^{-3} M; reaction time = 14 min. NB-Nitrobenzene; NA-Nitroaniline.

4. Calculation of the number of nanoparticles, of the number of surface gold atoms and of TOF

From the TEM experiments, we observed that AuNPs were spherical in shape, and we can roughly write:

$$V_{NP} = \bar{N} V_{atom} \qquad \textbf{(eq. 1)}$$

then

$$\left[\frac{4}{3}\pi (R_{NP})^3\right] = \bar{N}\left[\frac{4}{3}\pi (R_{met})^3\right] \qquad \textbf{(eq. 2)}$$

or, upon rearrangement

$$\bar{N} = f_{ov}\left(\frac{R_{NP}}{R_{met}}\right)^3 \qquad \textbf{(eq. 3)}$$

Here, V_{NP} is the volume of a nanoparticle (cluster), V_{atom} is the volume of an atom, R_{NP} is the radius of the NP, R_{met} is the metallic radius and $\overline{N}$is the total number of atoms within the NP and f_{ov} is the fraction of occupied volume which has a maximum value of 0.74 for a compact crystalline structure such as the bulky gold (face-centred cubic, FCC).

From eq. 3, and using the value of R_{NP} = 8 nm, obtained from TEM (AuNPs1% tea), and R_{met} = 144 pm, we determine the number of gold atoms per nanoparticle as N = 1.27×10^5.

The number of surface atoms, $\overline{N}_S$, on a unique gold nanoparticle was also estimated using the eq. 4, obtained[1] by dividing the surface area of the NP, by the cross section of each atom, ${\pi R_{met}}^2/f_{os}$ where f_{os} is the fraction of occupied surface which has a maximum value of 0.91 for a compact crystalline plane:

$$\overline{N}_S = 4 \times f_{os}\left(\frac{\overline{N}}{f_{ov}}\right)^{2/3} \qquad \textbf{(eq. 4)}$$

The number of AuNPs formed (N_{NP}) is given by the ratio of the total number of gold atoms, $N_{atom},$ over the number of gold atoms per nanoparticle, $\overline{N}$ (eq. 5)

$$N_{NP} = \frac{N_{atom}}{\overline{N}} \qquad \textbf{(eq. 5)}$$

considering

$$N_{atom} = \text{total number of moles of gold x } N_A \ \frac{\text{atoms}}{\text{mol}} \qquad \textbf{(eq. 6)}$$

and N_A, the Avogadro´s number (6.022 x 10^{23} mol^{-1}).

Hence, the total number (N_{ts}) of Au surface atoms (at the total surface of all the NPs, which are available for the catalytic reaction) is given by

$$N_{ts} = \bar{N}_S N_{NP} = \frac{4 \times f_{os}}{{f_{ov}}^{2/3}} \frac{N_{atom}}{\sqrt[3]{\bar{N}}} = \frac{4 \times f_{os} R_{met}}{f_{ov}} \frac{N_{atom}}{R_{NP}} = \quad \textbf{(eq. 7)}$$

This was further used for the calculation of the turnover frequency (TOF) (eq. 8).

$$TOF = \frac{M}{N_{ts} \cdot t} \quad \textbf{(eq. 8)}$$

where M is the number of molecules of the product (4-AP) produced during time t.

The results for all studied concentrations of AuNPs 1% tea extract are presented in Table S1.

Table S1 - Calculation of the number of AuNPs, of the number of Au surface atoms and of turnover frequency.[a]

Entry[b]	[Catalyst] (M)	N_{atom} x10^{-14} (eq. 6)	N_{NP} x 10^{-10} (eq. 5)	M x 10^{-14}[c]	N_{ts} x 10^{-13} (eq. 7)	t x 10 (h)	TOF (eq. 8)
1	3.0x10^{-7}	5.4	0.4	2.7	4.8	6.7	8.5
2	2.8x10^{-6}	50.6	3.99	64.2	44.4	3.3	43.9
3	8.0x10^{-6}	145	11.4	495	127	3	130
4	2.3x10^{-5}	415	32.7	639	364	2.3	76.3
5	3.2x10^{-5}	578	45.6	646	507	1	.127

[a] N_{atm} is the total number of gold atoms; N_{NP} is the number of gold nanoparticles formed; M is the number of molecules of the product (4-AP); N_{ts} is the total number of Au surface atoms and TOF is the turnover frequency. [b]Entries correspond to those of Table 1 with the same numbers. [c]Estimated from the conversion.

Reference

[1]Lewis, J.D.; Day, M. T.; MacPherson, V. J.; Pikeramenou Z., *Chem. Commun*. **2006**, 1433-1435.
